# Editorial: Environmental contaminants and animal health: Analysis, toxicity, and mitigation

**DOI:** 10.3389/fvets.2022.1102836

**Published:** 2022-12-08

**Authors:** Mohamed F. Abdallah, Wang Xu, Ahmed Abdeen

**Affiliations:** ^1^Department of Food Technology, Safety and Health, Ghent University, Ghent, Belgium; ^2^National Reference Laboratory of Veterinary Drug Residues, Ministry of Agriculture Key Laboratory for the Detection of Veterinary Drug Residues in Foods, Huazhong Agricultural University, Wuhan, China; ^3^Department of Forensic Medicine and Toxicology, Faculty of Veterinary Medicine, Benha University, Toukh, Egypt; ^4^Center of Excellence in Screening of Environmental Contaminants (CESEC), Benha University, Toukh, Egypt

**Keywords:** feed contaminants, renal toxicity, hepatic toxicity, mitigation, animal health, oxidative stress, omics

A wide range of environmental contaminants are potentially toxic to animals. This includes natural toxins (1, 2), pesticides (3), heavy metals (4) and other environmental contaminants such as microplastics (5). Animals are exposed to these pollutants mainly *via* consumption of contaminated feed and water, inducing variable deleterious effects including hepatotoxicity, nephrotoxicity, neurotoxicity, genotoxicity, immunosuppression, etc. Despite the great success in developing several tools for their detection, as well as investigating their toxic mechanisms, still many aspects remain unclear along with those related to the “emerging contaminants”. Moreover, in the last decade, the Toxicology field has become more interested in unraveling the combined toxicity of chemical mixtures under chronic exposure scenario. Finally, development of effective and realistic strategies regarding biomonitoring, remediation, and protective medication are still required to minimize the health hazards of such contaminants. This Research Topic “*Environmental Contaminants and Animal Health: Analysis, Toxicity, and Mitigation*” aimed at collecting papers, which implement cutting-edge techniques, to improve our knowledge and understanding on the toxic mechanisms of the commonly exposed (natural) toxins in (farm) animals and novel approaches to alleviate their harmful effects.

In this special e-collection, there are seven papers covering many aspects of the above mentioned topic. In Egypt, El-Sappah et al. tracked the presence of five trace elements (zinc, manganese, copper, cadmium, and lead) in Nile Tilapia (*Oreochromis niloticus*) from three different areas (Alexandria, Cairo and Aswan) as a bioindicator of environmental pollution. With the implementation of; (1) atomic absorption spectrophotometer analysis; (2) micronucleus frequency test; and (3) expression level of the Hsp70 gene assay, they concluded that the contents of cadmium, and lead were higher than the safe levels recommended by many international organizations such as FAO, WHO, and the European Commission. Indeed, more surveys are required to have a better insight into the accumulation of these metals in other aquatic organisms. On the other hand, Wei et al. performed a whole-transcriptome sequencing (RNA-seq) of liver samples from heifers to unravel the toxic effects due to a chronic exposure of cadmium present in feed. The obtained results showed several differentially expressed genes involved in autophagy regulation, apoptosis, lipid metabolism, anti-inflammation, and antioxidant enzyme activity. Doubtless to say, such findings are useful to predict the cadmium related toxicities and to propose appropriate treatments. Although the paper published by Sun et al. in this special issue was mainly focusing on the antibiotic resistance genes and its relation with the integrative conjugative elements, the used methodology could be followed in other toxin producing bacteria, such as *Clostridium botulinum, Listeria monocytogenes, Staphylococcus aureus*, and *Vibrio cholerae*, that represents an issue in terms of food and feed safety.

It is well known that selenium shows a unique capability as an antioxidant *via* scavenging free radicals and nano-selenium (NSe) poses antioxidant roles through the alleviation of the oxidative stress caused by heavy metal such as mercury, cadmium, lead, and other substances. Based on that, Du et al. explored the role of NSe against the cadmium-induced acute hepatic toxicity in male Kunming mice. Their published results show that NSe has a clear effect in reducing the toxic effect of cadmium by diminishing the generation of reactive oxygen species and activating the Nrf2 pathway. Several questions remain unanswered regarding the underlying mechanism of nano-selenium which acts against Cd-induced hepatotoxicity.

Aflatoxin B1 (AFB1) is the most natural carcinogenic substance in the history. This fungal toxin is a frequent contaminant of many agricultural commodities and it targets liver, kindly and other body organs. In chicken, the consumption of AFB1 contaminated feed may lead to a reduced growth performance and tremendous economic losses (1). Through their investigation to find a natural eco-friendly solution to reduce the toxic effect of AFB1 in broilers, Damiano et al. presented their findings on the protective effect on curcumin against the renal oxidative stress induced by the dietary exposure to low levels of AFB1. After 10 days of AFB1 exposure and curcumin, the known pathological effects due to AFB1 toxicity in broiler kidneys were not observed. It would be interesting to investigate the curcumin effect on a larger scale of broiler and formulate the curcumin as a feed supplement to commercialize it. Other natural substances were also studied for their protective effects in this special issue, showing the increasing interest of reporting safe products that are able to decrease the toxic effects of feed and food contaminants. Among these contaminants, acrylamide which is a widely used chemical material in several industries. Acrylamide represents a major threat to human health as it classified by IARC as human carcinogens. El-Shehawi et al. and Soliman et al. studied the protective effects of Taify Pomegranate juice and *Salsola imbricata* leaf extract against acrylamide, respectively. Results from experiments using male rats indicate that both substances are promising as antitoxic agents. This was supported by biochemical, real-time PCR, histopathological, and immunohistochemical analysis. An increase in levels of the endogenous antioxidative enzymes, including SOD, catalase, and GSH were significant after the exposure to either Taify Pomegranate juice or *Salsola imbricata* leaf extract. Additionally, anti-inflammatory effects through reduction of the inflammatory cytokines (TNF-α and IL-6) secretion and the enhancement of the inflammatory cytokine IL-10 level were recorded.

In summary, the results of the above mentioned studies represent an enormous amount of new relevant data on toxic mechanism of common environmental and feed contaminants as well as the potential of many natural substances to play a crucial role as protective agents against these toxic substances. However, there is still a long way to go in order to better understand the mechanisms of action and that makes our hopes on the young researchers and new technologies to enrich our knowledge in this discipline and other related fields.

## Author contributions

MFA wrote the editorial. All authors reviewed, edited, and approved the submitted version.
